# Correlating the ability of lignocellulosic polymers to constrain water with the potential to inhibit cellulose saccharification

**DOI:** 10.1186/s13068-014-0159-x

**Published:** 2014-11-19

**Authors:** Michael J Selig, Lisbeth G Thygesen, Claus Felby

**Affiliations:** IGN, Faculty of Science, University of Copenhagen, Rolighedsvej 23, 1958 Frederiksberg, Denmark

**Keywords:** Cellulose, Biomass recalcitrance, Entropy, Hemicelluloses, Water

## Abstract

**Background:**

Studies in bioconversions have continuously sought the development of processing strategies to overcome the “close physical association” between plant cell wall polymers thought to significantly contribute to biomass recalcitrance [Adv Space Res 18**:**251–265, 1996],[ Science 315**:**804–807, 2007]. To a lesser extent, studies have sought to understand biophysical factors responsible for the resistance of lignocelluloses to enzymatic degradation. Provided here are data supporting our hypothesis that the inhibitory potential of different cell wall polymers towards enzymatic cellulose hydrolysis is related to how much these polymers constrain the water surrounding them. We believe the entropy-reducing constraint imparted to polymer associated water plays a negative role by increasing the probability of detrimental interactions such as junction zone formation and the non-productive binding of enzymes.

**Results:**

Selected commercial lignocellulose-derived polymers, including hemicelluloses, pectins, and lignin, showed varied potential to inhibit 24-h cellulose conversion by a mix of purified cellobiohydrolase I and β-glucosidase. At low dry matter loadings (0.5% w/w), insoluble hemicelluloses were most inhibitory (reducing conversion relative to cellulose-only controls by about 80%) followed by soluble xyloglucan and wheat arabinoxylan (reductions of about 70% and 55%, respectively), while the lignin and pectins tested were the least inhibitory (approximately 20% reduction). Low field nuclear magnetic resonance (LF-NMR) relaxometry used to observe water-related proton relaxation in saturated polymer suspensions (10% dry solids, w/w) showed spin-spin, T_2,_ relaxation time curves generally approached zero faster for the most inhibitory polymer preparations. The manner of this decline varied between polymers, indicating different biophysical aspects may differentially contribute to overall water constraint in each case. To better compare the LF-NMR data to inhibitory potential, T_2_ values from monocomponent exponential fits of relaxation curves were used as a measure of overall water constraint. These values generally correlated faster relaxation times (greater water constraint) with greater inhibition of the model cellulase system by the polymers.

**Conclusions:**

The presented correlation of cellulase inhibition and proton relaxation data provides support for our water constraint-biomass recalcitrance hypothesis. Deeper investigation into polymer-cellulose-cellulase interactions should help elucidate the types of interactions that may be propagating this correlation. If these observations can be verified to be more than correlative, the hypothesis and data presented suggest that a focus on water-polymer interactions and ways to alter them may help resolve key biological lignocellulose processing bottlenecks.

## Introduction

The plant cell wall is a highly complex matrix of polymeric substances which predominantly includes crystalline cellulose, cross-linking glycans (hemicelluloses), pectins, and lignins [1–3]. In nature, these components are intertwined, providing structural support, osmotic control, and defense against pathogens aiming to utilize the same building blocks that industries target as second generation bioresources. Cellulose, hemicelluloses, pectins, and lignins are all differentially reactive, all adding their own biophysical barriers to their own degradation; furthermore, they all occlude access to each other [4–7]. In recent years this collective resistance and barrier to the plant cell wall as a resource has been aptly coined “biomass recalcitrance” [2,8].

Since 2005, the phrase “biomass recalcitrance” has been increasingly bestowed to describe the resistance of plant cell wall materials to enzymatic degradation processes utilized in current second generation bioconversion efforts. In research, most of this focus has strategized towards processes that overcome the tight association between cell wall polymers believed to significantly contribute to process bottlenecks. Despite much success in this area, only in recent years have bioconversion research efforts seen a gained interest in the elucidation of underlying biophysical mechanisms responsible for complicating substrate access and conversion rates. Along these lines, we have previously reported studies highlighting the organization of water in lignocellulosic systems and its contribution to processing bottlenecks such as the negative effects observed when operating at high dry matter loadings [9–11]. Here we transition from this work with a diversion into the biophysical aspects of polymer hydration, with the hypothesis that this may relate to the detrimental roles different plant cell wall polymers play with respect to the enzymatic conversion of cellulose by cellulases.

In an aqueous system, water molecules will associate with surfaces and become constrained as they form some level of organization at interfaces with non-water species, soluble or insoluble. Defining constraint, we imply here that associating water molecules become localized, more structured, and comparatively limited in available degrees of freedom, kinetic motion, and their ability to exchange with other water molecules as compared with water molecules in the bulk. When associating with polymers, water molecules can engage in weak (single or partial) or strong (double; bound to two groups) hydrogen bonding with polar groups on surfaces and localize in non-polar regions into unfavorable low-entropy states [12,13]. In addition, water molecules associated with surface bound/constrained water are generally thought to be at a greater level of organization or constraint than molecules in the bulk, although the extent to which this occurs is highly debated and often depends on the flexibility and static nature of the hydrated surface [9,14,15]. In all cases, this increased organization of water leads to lower states of entropy for the associated portions of the system’s water [16]. This becomes energetically unfavorable when the entropy change associated is not properly compensated by enthalpic components of interactions.

Where unfavorable states of water exist, there will be a tendency (or increased probability) for the system to change in order to reduce the presence of unfavorable (low-entropy) water and free it to the bulk. This is a basic component of hydrophobic interactions, which are key in events such as the binding of proteins to surfaces (productive or non-productive) and the formation of junction zones between like or dissimilar polymers [17]. In the plant cell wall, such interactions motivate the aggregation of amphiphilic glycan chains upon production that are then stabilized into crystalline cellulose by van der Waals interactions resulting in the energetically favorable formation of intermolecular carbohydrate-carbohydrate hydrogen bonds between neighboring chains [13]. Recent molecular dynamics studies indicate that such interactions play both non-productive and productive roles in enzymatic cellulose degradation processes. Examples include the energetically favorable binding of soluble gluco-oligosaccharides (greater than dimer) to crystalline cellullose and the potentially beneficial aggregation of oligomeric glycosylation sugars on the processive cellobiohydrolase I onto the hydrophobic face of cellulose [18,19].

Considering non-target substrates, numerous studies have reported the non-productive binding of enzymes to lignins as a contributor to stifled lignocellulose conversion rates [20,21]. Pareek and co-workers [22] have further shown that both xylan and glucomannan can also bind key cellulose-hydrolyzing enzymes. Additionally, xylooligomeric compounds are known to significantly inhibit cellulose hydrolysis [23], and Baumann and co-workers have even shown calorimetrically that long chain xylooligomers can non-productively bind key cellulase enzymes [24]. Can these polymers, other hemicelluloses, and even pectins bind in a similar manner to cellulose and other targets, occluding access to enzymes? Do they simultaneously interact non-productively with and sequester enzymes away from their target surfaces? If so, are there fundamental determinants for the potential for these detrimental interactions to occur with respect to each polymer?

**Figure 1:**
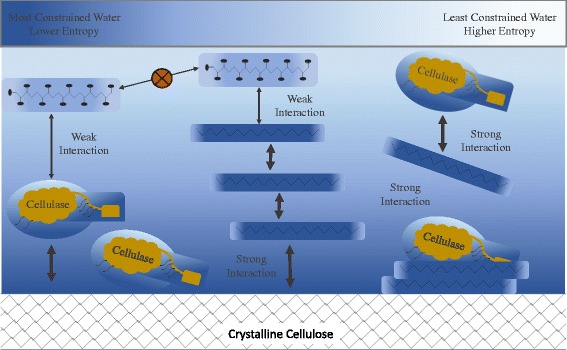
**Water constraint-biomass recalcitrance hypothesis.** Cartoon depiction of hypothesis stating that *the tendency for different plant cell wall polymers to be inhibitory to enzymatic lignocellulose conversion has a strong relation to the overall degree of constraint (or increased order, that is, lower entropy) these polymers put on water in a system by increasing the probability of detrimental interactions such as junction zone formation and the non-productive binding of key enzymes.* Structures shown are merely crude representations and do not accurately depict specific structures.

In consideration of these questions, we believe that a key fundamental determinant for the tendency of such interactions to occur may be related to the association of polymers with water that we have discussed above. Here we hypothesize that *the tendency for plant cell wall polymers to be inhibitory to enzymatic lignocellulose conversion is mechanistically related to the per-unit mass level of entropy-reducing constraint polymers put on the surrounding water which acts to increase the probability of said polymers to engage in detrimental binding interactions such as junction zone formation and the non-productive binding of enzymes*. This has been cartooned in Figure 1. In this context, the level of “constraint” per unit mass is simultaneously related to the degree to which water is constrained per unit of exposed surface area and the amount of exposed surface area per unit mass that a polymer presents to the surrounding water. Starting with these basic factors, the overall level of constraint will then be compounded or relieved by the overall flexibility of the polymer, and further compounded by pore spaces and structural features which physically confine water to partially enclosed spaces [15,25].

In this manuscript, we provide experimental support for our hypothesis by looking at the potential for a range of commercially available plant cell wall polymers to inhibit the saccharification of cellulose by a simplified cellulase system. We then relate this information to low field nuclear magnetic resonance (LF-NMR) relaxometry data (spin-spin, T_2_, relaxation time) used to observe the distribution and constraint of water in equally hydrated polymer preparations. In this data, free water molecules exhibit the longest relaxation times (about 3,000 ms), while water molecules that are more constrained by the lignocellulosic polymers exhibit shorter relaxation times. Relating this data to cellulase inhibition data, we have observed a general trend between the water-constraining and inhibitory nature of different cell wall polymers. The origins of this correlation do not appear to be specific to certain types of constrained water, since the distributions of constrained water pools vary considerably from polymer to polymer.

## Results and discussion

In this study we compare water-constraining (entropy-reducing) aspects of hydration to the inhibitory potential towards cellulose hydrolysis for a range of lignocellulosic polymers. Here, water constraint is assessed using LF-NMR, and it is operationally defined via spin-spin relaxation times. The polymers tested, with the exception of the lignin, are all complex carbohydrates. Typically lignins are thought of as being hydrophobic and carbohydrates hydrophilic, but in reality these macromolecular structures contain both hydrophilic and hydrophobic regions repeating to varying degrees across the polymer-water interface, and we would be better to think of them as existing along an amphiphilic spectrum. Furthermore, the placement on that spectrum can be a product of a number of chemical and conformational issues.

The association of carbohydrates with water will, for instance, be strongly affected by the orientation of polar hydroxyl groups on constituent sugars. This affects the level of intramolecular hydrogen bonding that occurs. Greater intramolecular bonding will reduce the total quantity of hydrogen bonded water molecules while simultaneously increasing the rigidity, static nature, and non-polar (hydrophobic) nature of the surface. These latter characteristics contribute to greater ordering of the surrounding water, a greater fraction of hydrogen bonded water molecules being strongly bound, and an increased prevalence of low-entropy low-density water zones along surfaces. Likewise, the absence of intramolecular hydrogen bonding leads to greater molecular flexibility and a greater abundance of weakly hydrogen bonded (less constrained single or partially bound) water molecules, which increases solubility but limits the ordering of the surrounding water [15,26,27]. It is important to keep in mind the complexity of issues such as this as we continue to investigate how the constrained nature of surface water influences binding interactions in simple and complex enzyme-plant polymer systems.

Prior to investigating two-polymer systems of plant cell wall polymers (cellulose plus an alternate polymer), we considered the case of a single-polymer system. Per unit surface area, the chemical character of a material will affect the degree to which individual surfaces constrain water, resulting in stronger or weaker binding interactions with other species in a system. Despite this, the simplest way that a material’s ability to constrain water can vary is through variations in accessible surface area. Holding the molecular structure constant, the larger the amount of surface area per unit mass, the greater the amount of water that is going to be associated with and constrained per unit mass of the polymer. This increased constraint is characterized in LF-NMR data by faster relaxing T_2_ relaxation curves.

**Figure 2:**
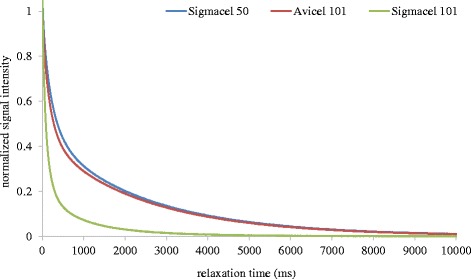
**LF-NMR of commercial celluloses.** Spin-spin, T_2,_ relaxation Carr-Purcell-Meiboom-Gill (CPMG) profiles for 10% (w/w) slurries of the commercial cellulose I preparations Avicel PH 101, Sigmacell 50, and Sigmacell 101.

**Figure 3:**
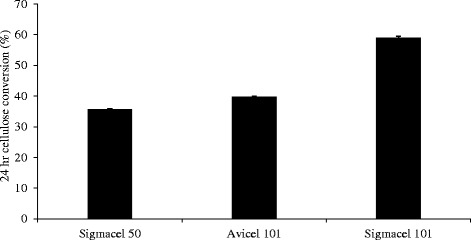
**Commercial cellulose saccharification.** 24-h enzymatic conversion of commercial cellulose preparations by a 10-mg/g cellulose loading of Novozymes Cellic CTec2 commercial cellulase.

In Figure 2 we show this by comparing relaxation curves for different commercial cellulose I preparations. From the literature both Avicel PH 101 and Sigmacell 50 have larger particle sizes and considerably higher crystallinity indices than the cellulose in the Sigmacell 101 preparation [28,29]. Both factors contribute to a greater surface area per unit mass for the Sigmacell 101, and hence more surface area to constrain associating water, which translates into the faster relaxing T_2_ curves in Figure 2 compared to the other celluloses. Furthermore, with accessible surface being a key factor in the efficiency of enzymatic cellulose degradation, it is not surprising that we observe (Figure 3) considerably higher 24-h extents of conversion for Sigmacell 101 by a set loading of commercial cellulase. However, we need to be aware that plant cell walls are more complex than these cellulose substrates and that the simple concept of surface area does not apply directly in systems with multiple lignocellulosic polymers that all differentially interact with water and each other.

**Figure 4:**
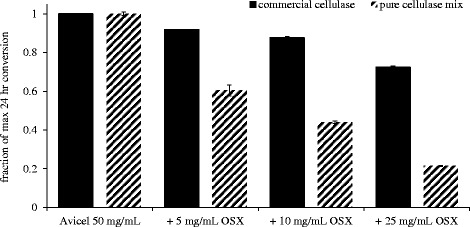
**Cellulase inhibition by oat spelt xylan.** Inhibition of cellulose hydrolysis by increasing addition of oat spelt xylan. Data is presented as a fraction of the maximum conversion achieved in the control cellulose-only hydrolysis.

Next, if we consider systems that include two cell wall polymers, the most obvious to study are cellulose-xylan systems. These two components have long been thought to be closely associated with one another despite not sharing covalent bonds, and recent studies have already pointed to the inhibitory potential of xylans and xylooligomers to both pure cellulases and cellulase cocktails [6,23,24]. In Figure 4, the inhibitory effects of xylan on cellulose hydrolysis are demonstrated even when small amounts relative to the cellulose (a 1-to-10 ratio) are present. This is particularly drastic with the cellobiohydrolase-only system where no xylanases are present to dismantle the interfering xylans. Furthermore, the inhibition of commercial cellulase here, which contains a modest xylan-degrading system, is in line with reports on the inhibitory effects of xylooligomeric compounds, which are often produced during the partial hydrolysis and solubilization of the less recalcitrant xylan fractions [30].

With respect to our water-constraint hypothesis, one might expect that dismantling large xylans (increasing the surface area) into small monomer and dimer sugars would drastically increase the constraint of water on the system and increase their inhibitory nature. In reality, the impact of hydrophobic aspects of the individual xylose units is much different at such a reduced degree of polymerization. Even for highly hydrophobic molecules there is a size breakpoint where the non-polar surface area is small enough to allow water molecules to circumvent the hydrophobic space and avoid the order-increasing (reduced entropy) disruption of the bulk-water hydrogen bond network that typically occurs in water surrounding larger hydrophobic molecules [17]. This point is reflected in LF-NMR data from our 2012 study on water availability in high dry matter lignocellulose conversion processes [11], which shows near order of magnitude faster relaxation times for similar concentrations of oat spelt xylan compared to monomer xylose.

**Figure 5:**
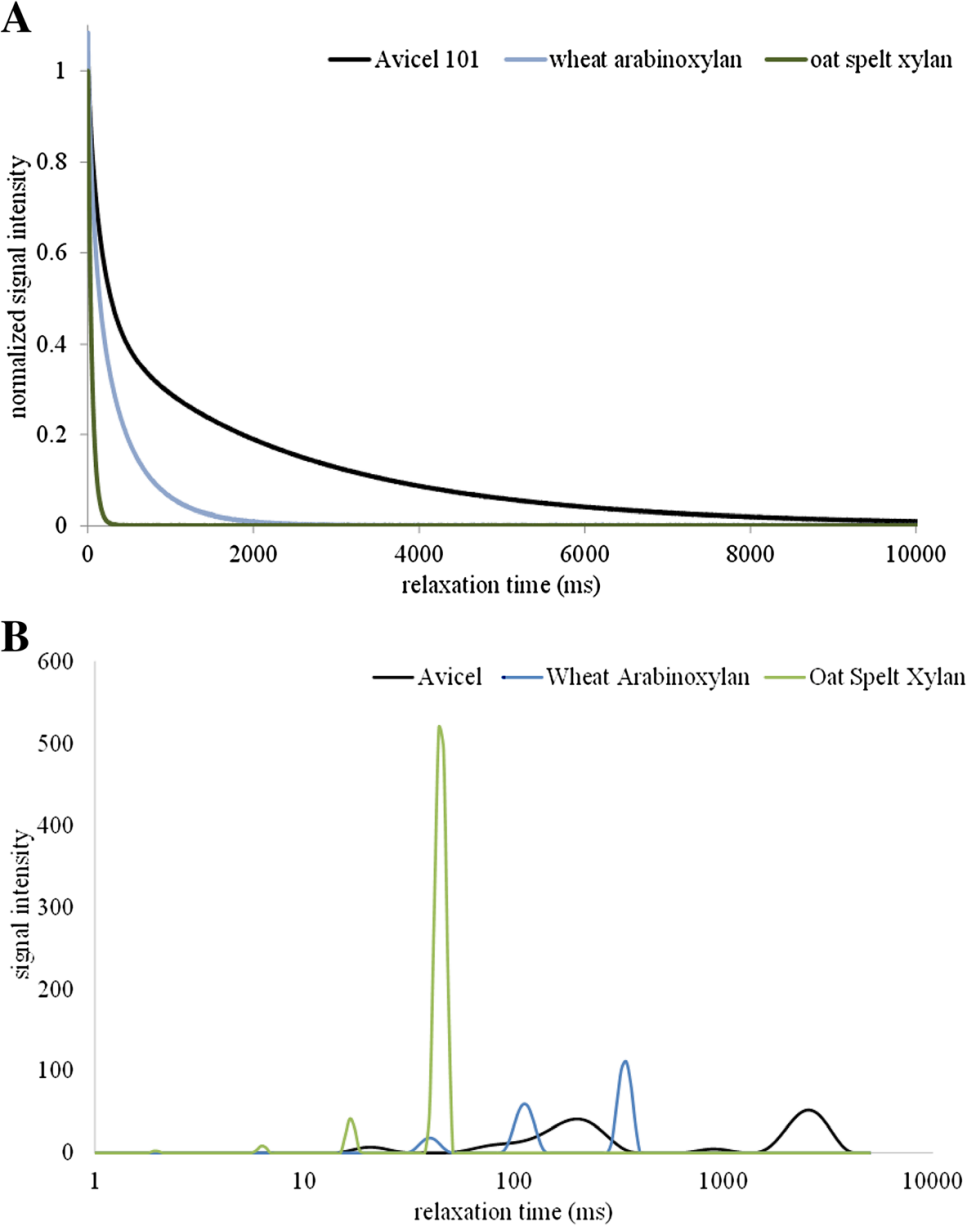
**Cellulose-xylan LF-NMR comparison.** T_2_ relaxation time data from low field NMR for Avicel, wheat arabinoxylan, and oat spelt xylan: **(A)** CPMG T_2_ relaxation curves; **(B)** CONTIN T_2_ distribution profiles. CPMG profiles are averaged profiles from triplicate experimental runs, and CONTIN distributions are single data sets representative of triplicate run data.

Continuing, while cellulose in the plant cell wall exists in a compact crystalline form, xylans are often heterogeneous and do not aggregate in an organized manner. This means that much more surface area on xylans is available for non-productive interactions than on crystalline cellulose for productive interactions. This translates into a greater ability for xylans to constrain water on a per-unit mass basis. The data in Figure 5A highlight this fact by comparing T_2_ relaxation curves for 10% (w/w) slurries of Avicel, soluble wheat arabinoxylan, and insoluble oat spelt xylan. The faster declining T_2_ relaxation curves associated with the xylans represent the fact that they constrain water to a greater degree than cellulose. Furthermore, we observed insoluble oat spelt xylan to be the most constraining; this is likely due to a greater level of branching in the arabinoxylan, particularly the highly flexible alpha-L-arabinofuranosyl side chains. These side groups, due to the flexible and fluid nature of their furanose ring structure and their inability to form intramolecular hydrogen bonds, bind more water but in a much weaker fashion compared to the unbranched xylan backbone. Unbranched xylan backbones tend to be more rigid due to the increased level of intramolecular hydrogen bonding among the xylose residues, and hence less soluble. The increased rigidity and exposure of both hydrophobic faces on the backbone more greatly constrain the surrounding water molecules and lead to a greater degree of disorganized binding interactions working to exclude this water and can lead to polymer precipitation [26,27,31].

To better visualize the differences in the distribution of water constraint among the different polymers, we look to previous studies on water organization by Felby and co-workers [9,11]. In these studies the CONTIN distribution of exponentials that contribute to the T_2_ relaxation curves (previously described by Provencher and co-workers [32]) were used to look at different pools of water in a system. These pools were used to generally categorize various states of constrained surface bound water, water constrained in pores and capillaries, and non-associated “free” water [9,33]. In Figure 5B these pools (associated CONTIN peaks) are shown to be drastically different between cellulose and the xylans discussed above, and likewise the xylan solubility appears to create even greater disparity. The profile for the Avicel 101 system shows a large pool around 3,000 ms, which would typically be designated as “free” water, while the profiles for the xylans indicate that all of the water is more constrained by the xylans in one form or another with little “free” water present.

**Figure 6:**
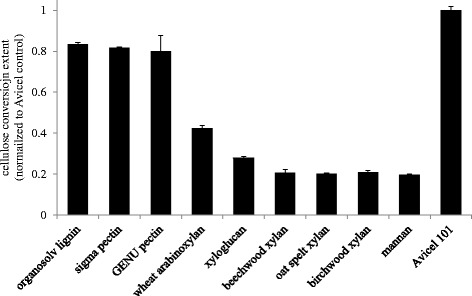
**Cellulase inhibition by lignocellulosic polymers.** Extent of maximum Avicel 101 control conversion achieved in the presence of 5-mg/mL loadings of each commercial plant cell wall component preparation.

Following this we investigated the potential for these xylans to inhibit the hydrolysis of cellulose by a simple cellulase mix and found that the insoluble oat spelt xylan was more inhibitory than the soluble wheat arabinoxylan. In Figure 6, it is shown that the oat spelt xylan reduced the effectiveness of the cellobiohydrolase-β-glucosidase mix by approximately 80% over 24 h compared to the cellulose-only control, while the wheat arabinoxylan reduced conversion extents by just over 50%. In line with our hypothesis, we believe the greater inhibitory potential is related to the greater amount of water constraint imparted by the oat spelt xylan as discussed above, as well as the resultant binding interactions that lead to the xylan’s insolubility. Furthermore, expanding the inhibition study to include other commercial plant cell wall polymer preparations we found that all of the insoluble hemicelluloses tested were the most inhibitory, while the soluble carbohydrates were less so; the α-linked pectins being less inhibitory than the soluble hemicelluloses (wheat arabinoxylan and tamarind xyloglucan). Furthermore, the organosolv lignin preparation, despite reducing conversion by about 20%, was the least inhibitory.

**Figure 7:**
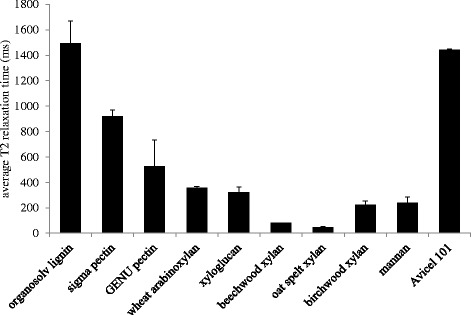
**Comparative LF-NMR for commercial lignocellulosic polymers.** Data T_2_ relaxation times from monocomponent exponential fit of CPMG profiles. Data represent averages of triplicate analyses.

To investigate our initial hypothesis, the inhibition experimental data presented in Figure 6 were then followed up with a series of LF-NMR runs on 10% (w/w) slurries of the same commercial cell wall polymer preparations. The spin-spin T_2_ relaxation time data was used to compare the constraint the preparations put on the water to the inhibition data. In general, the relaxation curves correlated well with the inhibition data. The more inhibitory compounds produced faster relaxing curves and the less inhibitory compounds displayed slower relaxing curves. A comparison of all relaxation curves on a single plot proved quite cumbersome, with curves crossing one another at different points due to the different mechanisms with which each compound may constrain water. To discombobulate the matter, T_2_ relaxation times from monocomponent exponential fits of each curve were used to provide a single measure of the total water constraint in the water-polymer systems; here, the lowest relaxation times represent the greatest constraint. The average relaxation times from these exponential fits are presented in Figure 7 and generally decline in line with the inhibitory potential of the polymer preparations presented in Figure 6.

**Figure 8:**
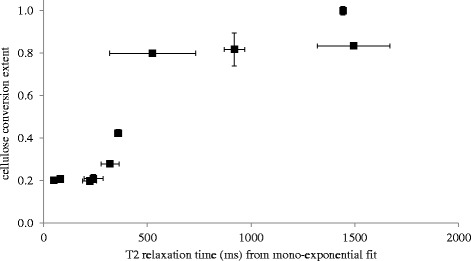
**Cellulase inhibition versus T**_**2**_ **relaxation time.** Fraction of achieved control conversion extent versus T_2_ relaxation time from monoexponential decay for cellulose hydrolyses inhibited by various lignocellulosic polymers.

**Figure 9:**
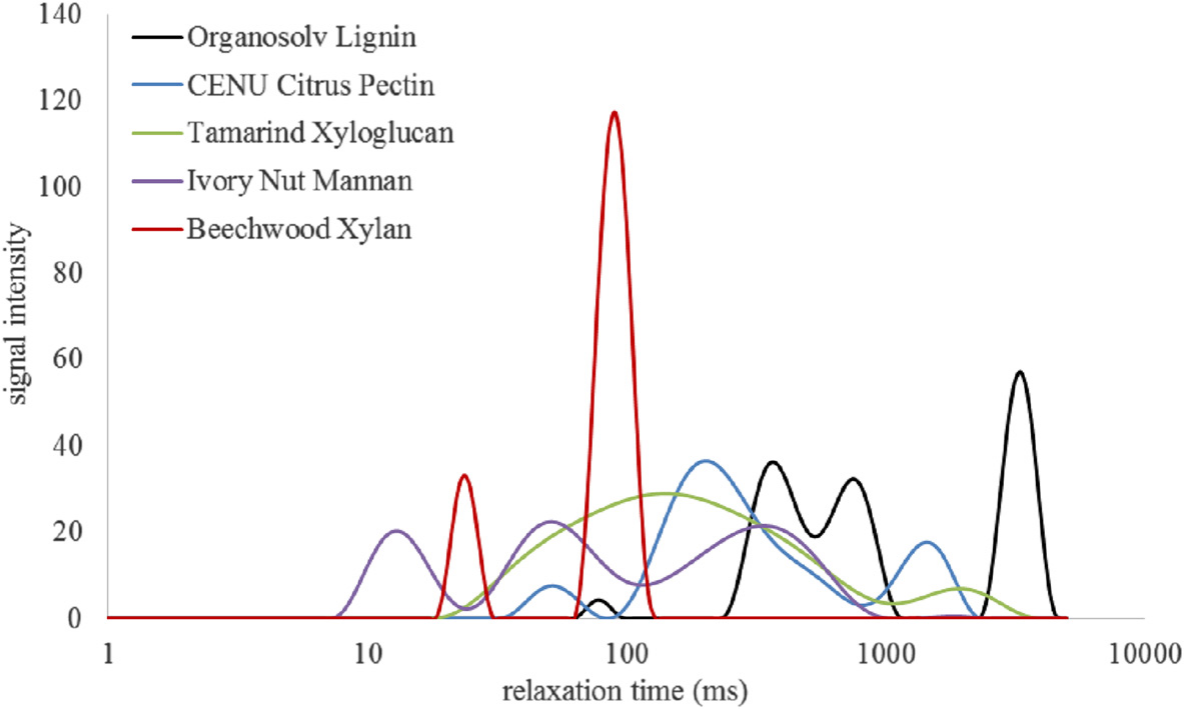
**CONTIN T**_**2**_ **distribution profiles.** For 10% (w/w) slurries of organosolv lignin, citrus pectin, tamarind xyloglucan, ivory nut mannan, and beechwood xylan. CONTIN distributions are single data sets representative of triplicate run data.

In Figure 8 a plot comparing the inhibition data and the averaged T_2_ data is presented to better visualize the general relationship. The data appear to correlate well, but there is not a perfect direct relationship between the two factors. This was not expected either, as there are many aspects of polymer hydration that potentially contribute to either data point for this broad array of amphiphiles. This notion is well illustrated in Figure 9, which includes the CONTIN T_2_ distribution profiles on a handful of the tested polymers. From this figure it is clear that all carbohydrates tested constrain all of the slurry water to some degree, since none have significant peaks at the 3,000 ms relaxation time indicative of free water. Despite this, all four carbohydrates (and the xylans in Figure 5) clearly constrain the water in different ways, as the peak distributions for all samples shown here are entirely different, even when some of the samples produced identical monocomponent T_2_ values. Meanwhile, for the organosolv lignin sample a considerable free-water peak exists, which we speculate suggests that this lignin sample presents a significantly reduced surface area-to-mass ratio compared to the carbohydrates. We further suggest that the limited available lignin surface area that can constrain water may limit its ability to engage in negative hydrophobically driven binding interactions, despite the highly hydrophobic nature of the material.

Of the polymer set studied, the data do highlight the critical nature of hemicellulose removal from lignocelluloses if cellulose is the primary conversion target. Also, the fact that the most water-constraining hemicelluloses were all insoluble hemicelluloses does a nice job of displaying how changes increasing the static nature of polymers can do a lot to increase the organization of water surrounding them. Furthermore, the minor effects of the lignin observed here add weight to the debate on the detriment lignin poses in lignocellulose conversion processes. It gives favor to arguments that lignin, while it may be inhibitory, is less problematic when considering all of the polymers present in the plant cell wall [34]. However, it is worth noting that we have only canvassed a small pool of specific isolated variants of common cell wall polymers. Since lignins, pectins, and hemicelluloses can occur or be isolated into a range of sizes and chemical and structural conformations, our data here should not dictate a concrete order with respect to the inhibitory nature or water-constraining nature of plant cell wall polymer classes in all situations (that is, all xylan forms may not always be more inhibitory than all lignin forms).

## Conclusions

We have put forth a hypothesis that the inhibitory potential of polymers within the plant cell wall to cellulose hydrolysis is related to the degree to which the polymers constrain the surrounding water per unit mass. This hypothesis is supported with data correlating the inhibition of a simple cellulase system by different cell wall polymers to low field NMR T_2_ relaxation time data that has been used to gauge overall water constraint in prepared polymer slurries. If the observed relationship between water constraint and polymer inhibitory potential proves to be more than a correlation, then investigation into methods altering water polymer organization may lead to breakthroughs regarding problematic bottlenecks in lignocellulose processing. Investigation into whether the inhibitory effects related to this correlation are dominated by enzyme-polymer non-productive binding events or steric hindrances resulting from polymer-polymer junction zone formation should provide insight on the matter. Overall, the study highlights the need to properly understand water-polymer interactions in lignocellulose processing. Continued work in this area has the potential to paint a clearer picture of the fundamental mechanisms driving key industrial bottlenecks and may lead to cost-effective methods for circumventing them.

## Materials and methods

### Plant cell wall carbohydrates

The primary cellulose preparation used for all inhibition studies was Avicel PH 101 (11365 FLUKA; Sigma-Aldrich, St. Louis, MO); other cellulose preparations used included Sigmacell 50 (S5504) and Sigmacell 101 (S6790; Sigma-Aldrich). Hemicellulose preparations used included oat spelt, birchwood, and beechwood xylans from Sigma-Aldrich, as well as wheat arabinoxylan (low-viscosity; P-WAXYL), tamarind xyloglucan (P-XYGLN), and ivory nut mannan (P-MANIV) from Megazyme International (Wicklow, Ireland). Pectins included in this study were citrus pectin (Sigma-P9135) from Sigma-Aldrich and GENU Pectin (citrus) from CP Kelco (Atlanta, GA). The lignin source for this study was organosolv lignin (371017 ALDRICH) obtained from Sigma-Aldrich.

### Enzymatic saccharifications

Enzymatic saccharifications were performed at 48°C in 50 mM sodium citrate buffer, pH 4.8, with initial dry cellulose concentration solids set at 1.0% (w/w) in 2.0-mL Eppendorf tubes. All hydrolyses were run for 24 h. In the initial experimentations where commercial cellulase was used, Novozymes’ (Bagsværd, Denmark) Cellic CTec2 cellulase was loaded at 10 mg protein/g cellulose. All remaining inhibition studies utilized a simple cellulase mix consisting of cellobiohydrolase I (*Trichoderma longibrachiatum;* Megazyme International; E-CBHI) and β-glucosidase (*Aspergillus niger*; Megazyme International; E-BGLUC) loaded at 4 and 1 mg protein/g cellulose, respectively. All inhibition study saccharifications were run with Avicel PH 101 loaded at 1% (w/w) initial dry solids and the additional cell wall component loaded at 0.5 % (w/w) initial dry solids. Control digestions of the added cell wall components (without cellulose) showed no significant release of glucose from the substrates. All conversion extents for the inhibition runs were normalized to the conversion extent of the cellulose-only control for ease of comparison (glucose release from inhibited cellulose hydrolysis/average glucose release from cellulose-only control; percent reduction in conversion is calculated as 100 × [control conversion-inhibited conversion]/control conversion). All saccharifications were replicated in triplicate.

### Low field nuclear magnetic resonance (LF-NMR) relaxometry

Low field nuclear magnetic resonance (LF-NMR) relaxometry was performed on a Bruker (Billerica, MA) mq20 minispec with a 0.47-T permanent magnet equivalent to a 20-MHz proton resonance frequency held at a constant 40°C. Spin-spin, T_2_, relaxation times were determined using the Carr-Purcell-Meiboom-Gill (CPMG) sequence for all runs. Thirty-two scans were acquired on a 5-s recycle delay; for each scan 8,000 echoes were collected with pulse separations of 0.7 ms for scans at respective initial dry solids loadings of 30 and 10% (w/w). The CONTIN Laplace transformation method outlined previously [32] was used to determine T_2_ relaxation time distributions from the CPMG data. All NMR data presented are for 10% (w/w) dry solids suspensions of selected commercial plant cell wall polymer preparations; triplicate suspensions of each preparation were prepared and pre-equilibrated for 30 min at 40°C prior to NMR analysis. All CPMG and CONTIN profiles presented are average data sets obtained from these triplicate runs.

### HPLC

Glucose concentration was used as a measure of saccharification efficiency in all enzymatic experiments. Concentrations of glucose in all hydrolysates were determined on an UltiMate 3000 system from Dionex (Sunnyvale, CA) with a Rezex ROA-Organic Acid H + column (Phenomenex; Torrance, CA) running 0.5% sulfuric acid at 0.6 mL/min as the eluent.

## Abbreviations

CPMG: Carr-Purcell-Meiboom-Gill
LF-NMR: Low field nuclear magnetic resonance
